# A proteomic classifier panel for early screening of colorectal cancer: a case control study

**DOI:** 10.1186/s12967-024-04983-5

**Published:** 2024-02-21

**Authors:** Hanju Hua, Tingting Wang, Liangxuan Pan, Xiaoyao Du, Tianxue Xia, Zhenzhong Fa, Lei Gu, Fei Gao, Chaohui Yu, Feng Gao, Lujian Liao, Zhe Shen

**Affiliations:** 1 Department of Colorectal Surgery (H.H), and Department of Gastroenterology (C.Y. and Z.S.), College of Medicine, The First Affiliated Hospital, Zhejiang University, Hangzhou, 310006 Zhejiang China; 2 Durbrain Medical Laboratory, Hangzhou, 310000 Zhejiang China; 3 Changzhou Wujin People’s Hospital, Changzhou, 213000 Jiangsu China; 4 Department of General Surgery, School of Medicine, Shanghai Tenth People’s Hospital, Tongji University, Shanghai, 200072 China; 5 Shanghai Key Laboratory of Regulatory Biology, School of Life Sciences, East China Normal University, Shanghai, 200241 China

**Keywords:** Colorectal cancer, Early detection, Protein biomarker, Mass spectrometry

## Abstract

**Background:**

Diagnosis of colorectal cancer (CRC) during early stages can greatly improve patient outcome. Although technical advances in the field of genomics and proteomics have identified a number of candidate biomarkers for non-invasive screening and diagnosis, developing more sensitive and specific methods with improved cost-effectiveness and patient compliance has tremendous potential to help combat the disease.

**Methods:**

We enrolled three cohorts of 479 subjects, including 226 CRC cases, 197 healthy controls, and 56 advanced precancerous lesions (APC). In the discovery cohort, we used quantitative mass spectrometry to measure the expression profile of plasma proteins and applied machine-learning to select candidate proteins. We then developed a targeted mass spectrometry assay to measure plasma concentrations of seven proteins and a logistic regression classifier to distinguish CRC from healthy subjects. The classifier was further validated using two independent cohorts.

**Results:**

The seven-protein panel consisted of leucine rich alpha-2-glycoprotein 1 (LRG1), complement C9 (C9), insulin-like growth factor binding protein 2 (IGFBP2), carnosine dipeptidase 1 (CNDP1), inter-alpha-trypsin inhibitor heavy chain 3 (ITIH3), serpin family A member 1 (SERPINA1), and alpha-1-acid glycoprotein 1 (ORM1). The panel classified CRC and healthy subjects with high accuracy, since the area under curve (AUC) of the training and testing cohort reached 0.954 and 0.958. The AUC of the two independent validation cohorts was 0.905 and 0.909. In one validation cohort, the panel had an overall sensitivity, specificity, positive predictive value (PPV), and negative predictive value (NPV) of 89.9%, 81.8%, 89.2%, and 82.9%, respectively. In another blinded validation cohort, the panel classified CRC from healthy subjects with a sensitivity of 81.5%, specificity of 97.9%, and overall accuracy of 92.0%. Finally, the panel was able to detect APC with a sensitivity of 49%.

**Conclusions:**

This seven-protein classifier is a clear improvement compared to previously published blood-based protein biomarkers for detecting early-stage CRC, and is of translational potential to develop into a clinically useful assay.

**Supplementary Information:**

The online version contains supplementary material available at 10.1186/s12967-024-04983-5.

## Background

Colorectal cancer (CRC) is a leading cause of cancer-related deaths worldwide. In the United States alone, CRC is the second most common cancer with a mortality rate ranks the second among all cancers [1]. The outcome of CRC patient is generally poor, to a large extent due to lack of effective testing methods to detect tumors at early stages. If tumor is detected at precancerous or localized stage, 90% of CRC patients can survive more than five years. Whereas if it is diagnosed at stages IIb or later, the survival rate is dramatically lower, primarily due to complications associated with tumor spreading and metastasis [2]. Currently, the “gold standard” of clinical procedure for diagnosing CRC is the colonoscopy [3], during which the tumors can be removed and pathologically examined. There are also a number of stool- and blood-based non-invasive screening methods to aid the detection of CRC at early stages [4]. The FOBT/FIT uses immunoassay to measure the hemoglobin in the stool [5]. Two DNA tests measure the methylation status at the promoter region of mSEPT9 or SDC2 gene in the blood and the stool respectively [6, 7]. Immunoassays to measure the traditional tumor antigens including carbohydrate antigen CA19-9 and carcinoembryonic antigen (CEA) [8, 9] are widely used in the clinic. In recent years, the burgeoning field of liquid biopsy has made great strides to non-invasive test of multiple cancer types at early stages, including CRC [10–12]. The assay detecting septin9 gene methylation in the blood has offered a laboratory developed test (LDT, Epi procolon) [13]. Another LDT combines FIT and detecting BMP3/NDRG4 DNA methylation as well as KRAS mutation in the fecal sample (Cologuard) [14]. These exciting developments have opened up new opportunities for early detection of CRC.

However, there are still windows for improvement in terms of sensitivity and specificity as well as other issues such as cost-effectiveness and patient compliance. The FOBT/FIT test yields a sensitivity of 73.8% with a specificity of 95% [14], whereas the protein biomarkers provide a sensitivity of 80% [15]. Epi procolon test has a specificity of 88%, yet the sensitivity is 87% for early-stage CRC [13]. Cologuard has a sensitivity of 92.3%; its specificity falls to 86.6% [14]. Improving both the sensitivity and specificity simultaneously can screen CRC with much improved accuracy, limiting both the false positive and false negative calls. On the other hand, protein markers in clinical use such as CEA and CA19-9 is far from sensitive. Although colonoscopy is the “gold standard”, the invasive nature raises the issue of patient compliance. Therefore, a non-invasive, sensitive and specific screening method that detects and diagnoses CRC at the earliest stage is needed.

Advanced precancerous lesions (APC), including advanced adenomas and large sessile serrated polyps (greater than 1 cm in size), are considered to have a high probability of developing into fully grown cancers [16]. Nevertheless, the progression time window of advanced adenomas can be as long as ten years, providing both an opportunity and a challenge to detect these lesions in a timely manner [16, 17]. The standard for detecting advanced adenomas is also optical colonoscopy [18]. Due to patient compliance and rare complications, applying colonoscopy as a general screening method to detect APC remains to be a challenge [16]. Although liquid biopsy based on gene test to detect adenomas has been developed, it still suffers from low sensitivity [14]. Therefore, developing much improved assays to detect precancerous lesions is also highly desirable.

Currently, mass spectrometry technologies have achieved high sensitivity and data acquisition speed. Whereas traditional data-dependent acquisition (DDA) provides straightforward protein identification and ease of data interpretation, data-independent acquisition (DIA) dramatically expanded the dynamic range of quantitation [19, 20]. Multi-reaction monitoring (MRM), another mass spectrometry technology widely used in pharmaceutical industry, is well suited to accurately quantify hundreds of peptides in one experiment [21, 22]. As such, we applied both DDA and DIA to investigate the plasma proteome in patients diagnosed with CRC and APC. Our analysis captured unique molecular features of the plasma proteome in these conditions. We further developed an MRM assay combined with machine learning to discover and validate a panel of protein biomarkers to distinguish CRC from benign polyps and from healthy subjects.

## Methods

### Human samples

Plasma samples of the Chinese population were collected from three hospitals between September 2020 and September 2022. Peripheral venous blood samples were collected before any treatment procedure, and were centrifuged at 500 × g for 10 min to obtain plasma within two hours and stored at − 80 ℃. All plasma samples were transported to the central lab by cold chain system at − 80 ℃ and stored at − 80 ℃ before MS experiments.

In the discovery cohort, plasma samples from 70 patients diagnosed with colorectal cancer (CRC) and 72 healthy subjects were collected from Shanghai Tenth People’s Hospital, Tongji University School of Medicine. In the validation cohorts, the plasma samples were collected from Shanghai Tenth People’s Hospital, the First Affiliated Hospital of Zhejiang University and Changzhou Wujin People’s Hospital, respectively. We obtained written informed consent from each participant.

We excluded patients with any malignant tumors within five years prior to current diagnosis. The inclusion criteria were as follows: the age of all subjects is over 40 years (inclusive), with a balanced gender distribution; in the group of healthy subjects there should have no evidence of malignant tumor nor colorectal neoplasm; in the CRC group all diagnosis was confirmed by pathological evidence; in the APC group all patients went through colonoscopy and the diagnosis of either advanced adenomas or sessile serrated polyps were made by an experienced pathologist.

### Processing of plasma samples

For the two discovery cohorts, the plasma samples were processed to deplete the top 14 high-abundant proteins (Cat. # A36370, Thermo Science, USA), and protein concentration was determined using the BCA kit (Cat. # P0012, Beyotime, China). The 14 proteins included Albumin, IgA, IgD, IgE, IgG, IgG (light chains), IgM, Alpha-1-acid glycoprotein, Alpha-1-antitrypsin, Alpha-2-macroglobulin, Apolipoprotein A1, Fibrinogen, Haptoglobin, and Transferrin. From each sample, 25 μg protein was suspended in 50 mM NH_4_HCO_3_ solution. The proteins were treated with 10 mM DTT at 95 °C for 10 min and alkylated with 15 mM iodoacetamide (Cat. # I1149, Sigma Aldrich, USA) in the dark for 30 min. Then the protein was digested with sequencing grade trypsin (1:50, Cat. # V5113, Promega) overnight at 37 °C. The resulting peptides were desalted with a 96-well SOLA solid-phase extraction apparatus and vacuum dried. The peptides were stored in a freezer at − 80 ℃. and ready for mass spectrometry analysis.

For the four validation cohorts, the plasma samples were processed for mass spectrometry analysis without depleting the high-abundant proteins and the rest of experiment procedure was identical to the discovery cohorts.

### LC–MS/MS analysis of plasma samples using DDA and DIA

The plasma protein digests were analyzed using an EASY-nLC1000 liquid chromatography coupled with an Orbitrap Exploris™ 240 mass spectrometer (Thermo Fisher Scientific, USA). Peptides were resuspended in buffer A (2% ACN, 0.1% formic acid) and spiked with indexed retention time (iRT) peptides (Omicsolution, China). The iRT peptides are a set of standard peptides used in DIA experiments for high-accuracy calibration of chromatographic elution time, so as to improve reproducibility of MS experiments across laboratories. An equivalent to 2 µg of protein digest from each sample was loaded onto a C18 column (Cat. # 164534, Thermo Scientific, USA) linked with a pre-column (Cat. # 164535, Thermo Fisher Scientific, USA) and separated at a flow rate of 250 nL/ min. The mobile phases consisted of buffer A and buffer B (98% ACN, 0.1% formic acid). A 90 min gradient from 1 to 8% buffer B in 1 min, 8% to 28% in 71 min, 28% to 40% in 9 min, 40% to 100% in 2 min, and 100% for 7 min was used. For DIA analysis, peptides were resuspended in buffer A and spiked with iRT peptides. 1.5 µg of protein digest from each sample was loaded onto the C18 column.

The mass spectrometry was operated in positive mode in all cases. For DDA analysis, the nano-electrospray was operated using the ion transfer tube with a temperature setting of 275 °C. One full scan MS from 400 to 1400 m/z followed by 12 MS^2^ scans were cycled throughout the entire MS experiment. MS spectra were acquired with a resolution of 70000 with a maximum injection time (IT) of 60 s and an automatic gain control (AGC) target value of 3e6. MS^2^ spectra were obtained in the higher-energy collisional dissociation (HCD) mode with an isolation window of 1.6 m/z, using a normalized collision energy of 27%, resolution at 17500 with a maximum injection time of 50 s and an AGC target of 5e5. Centroid mode was used to collect both the MS and MS^2^ spectra.

For DIA analysis, the ion transfer tube was operated with a temperature setting of 320 °C. MS spectra were acquired with a resolution of 60000 with a maximum injection time (IT) of 120 ms and an AGC target value of 3e6. Isolation window for MS^2^ was set to 20 Da for the mass range 350–400 m/z, 9 Da window for the mass range 400–800 m/z, 12 Da window for the mass range 800–1000 m/z, and 25 Da window for the mass range 1000–1200 m/z.

### Multiple reaction monitoring quantitation of plasma proteins

Concentrations of target proteins in the plasma were measured using MRM on a QTRAP 5500 mass spectrometer (Sciex, USA) equipped with a turbo v ion source (Sciex, USA). The instrument parameters of the MRM assay were optimized for each synthetic peptide by directly infusing the peptides into the mass spectrometer. The top three high-intensity product ions of each peptide precursor ion were selected based on the optimal collision energy (CE) values and collision cell exit potential (CXP). All optimized data were collected and compared to theoretical spectra, and three high-intensity y-ions were used for subsequent MRM assays.

The peptides were separated using an LC-20AD (SHIMADZU, Japan) liquid chromatographic system. Buffer A was 0.1% formic acid in distilled water and buffer B was 0.1% formic acid in 98% acetonitrile. Peptides were reconstituted in buffer A and 15 µL of each sample was loaded into the sample loop. A gradient consisting of 6% buffer B for 2 min, 6–28% buffer B for 16 min, 28–98% buffer B for 0.5 min, 98% buffer B for 3 min, 98– 6% buffer B for 0.5 min, and 6% buffer B for 3 min was used. The MS detection was carried out in positive mode with the following parameters: electrospray voltage of 5500 V, curtain gas at 40 psi, ion source gas 1 (GS1) at 55 psi, ion source gas 2 (GS2) at 55 psi, and temperature at 500 °C. Quantitation were performed using the scheduled MRM mode. The time of MRM detection window was 180 s, and the cycle time was 1.0 s. The mass spectrometer was controlled by the Analyst software (Sciex, USA).

### Mass spectrometry data analysis

The DDA spectra were searched using Protein Discoverer 2.4 (Thermo Fisher Scientific, USA) against a UniprotKB human database (UP000005640). The search parameters were set as the following: trypsin was set to the protease type and two missed cleavages were allowed, the precursor mass tolerance was set to 10 ppm, and the fragmentation ion mass tolerance was set to 0.02 Da. The false discovery rate (FDR) was set to 1% at both the peptide and protein level. The maximum number of variable modifications was set to two.

The DIA spectra were searched using the DIA-NN (version 1.7.15) software [23] with a UniprotKB human database (UP000005640). The precursor mass tolerance was 10 ppm; trypsin was set as the protease and two missed cleavages were allowed. The maximum number of variable modifications was set to three. The precursor mass range was from 350 to 1250 m/z, and the fragmentation ion mass range was from 100 to 2000 m/z. The false discovery rate (FDR) was set at 1% at the peptide level.

### GO and KEGG pathway analysis

GO and KEGG enrichment analysis were performed using bioinformatics resources including Metascape (https://metascape.org/) and David v6.8 (https://david.ncifcrf.gov/). R package ClusterProfiler [24] was applied to generate the graphs.

### Feature selection and logistic regression

The mean decrease of Gini index (MDG) was calculated in a random forest feature selection model for both the DDA and DIA data. Gini index is a measurement of variance in random forest algorithm, in which lower variance and thus lower Gini index results in more accurate classification. We selected top 40 proteins based on the MDG values. To further narrow down the list of biomarker candidates from these 40 proteins, we applied the following criteria: 1. These proteins are differentially expressed in both datasets. 2. Literature reported cancer biomarkers were given priority for consideration.

The expression values of the peptide surrogates for the candidate proteins were used to build a logistic regression model to classify subjects as either healthy or colorectal cancer patients. The finalized panel of proteins were validated by new patient cohorts.

### Statistical analysis

R (version 4.2.2) was used for all the statistical analyses, including data preprocessing, differential expression analysis, volcano plot, and principal component analysis (PCA). For differential expression analysis, p value < 0.05 were considered statistically significant and fold change of > 1.25 or < 0.80 were considered up- or down-regulated, respectively. We utilized a more subtle fold change criterion in order to obtain more potential protein markers in the discovery phase, and further validated them in target validation phase. Proteins with more than 50% missing values were removed. The distribution of protein expression was tested for normality across all samples; then, t-test was applied for those with normal distribution, while Wilcoxon ranked sum test was performed for those failed to pass the normality test.

The discovery phase of the study was designed to obtain a statistical power of 85%, a one-sided type I error rate of 0.05, and a median effect size. With these parameters, the calculated sample size is 59 healthy subjects and 59 colorectal cancer patients, respectively.

## Results

### Study design

**Fig. 1:**
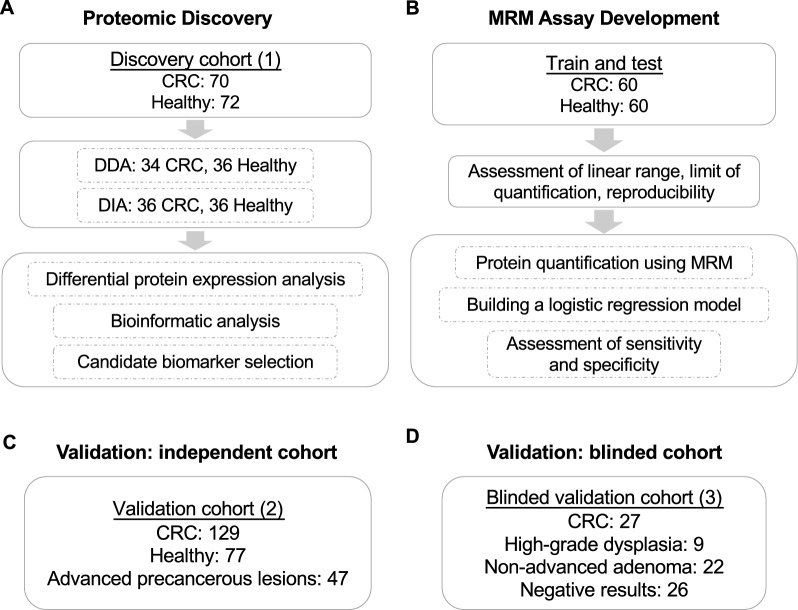
Study design. Flow chart showing the discovery and validation phases of this study. **A** Discovery cohort for quantitative proteomic analysis using DDA and DIA methods. **B** MRM targeted proteomic assay development. **C** Applying the MRM assay to an independent validation cohort of patients to classify CRC and APC patients from healthy controls. **D** Applying the MRM assay to a blinded validation cohort, in which the diagnosis was disclosed only after the test results were given

The design of this study is illustrated in Fig. 1. In the discovery phase, we enrolled a total of 142 subjects (including 70 CRC patients and 72 healthy controls, cohort 1) from one hospital. The plasma samples from half of the discovery cohort were analyzed using DDA method, whereas the other half were analyzed using DIA method. To monitor the data acquisition process, we interspersed quality control (QC) samples during mass spectrometry data acquisition, which composed of small portion of plasma samples from all patients in the discovery cohort (Fig. 1A). In the assay development phase, MRM method was applied to part of the samples from cohort 1 to measure the plasma concentration of selected protein biomarkers based on the signature peptides. In the meantime, MRM assay were developed to assess the linear range, limit of quantification, and reproducibility (Fig. 1B). The validation phase included 129 CRC patients and 77 healthy controls from two different hospitals as well as 47 cases of APC as cohort 2 (Fig. 1C). We then enrolled another independent, blinded validation cohort of patients as cohort 3 from the third hospital, in which the laboratory did not know the diagnosis of the patients until the blood test results were complete (Fig. 1D). In all cohorts, cancer patients either went through colonoscopy or surgery and the tumor tissues were pathologically confirmed. The demographic data for the patients enrolled in the discovery cohort is shown in Table 1. The demographic data for all the patients enrolled in validation cohorts is shown in Additional file 6: Table S1.

**Table 1:** Demographic data and characteristics of the participants in the discovery cohort

		DDA (n = 70)			DIA (n = 72)		
Characteristics	Level	HC	CRC	P	HC	CRP	P
Number		36	34		36	36	
Gender	Female	21 (58.3)	11 (32.4)	0.034	16 (44.4)	14 (38.9)	0.811
	Male	15 (41.7)	23 (67.6)		20 (55.6)	22 (61.1)	
Age (mean (SD))		53.61 (4088)	61.09 (8027)	< 0.001	53.11 (5.87)	60.78 (8.78)	< 0.001

### Quantitative mass spectrometric analysis of plasma proteome using two different data acquisition methods on independent batches of patient samples

**Fig. 2:**
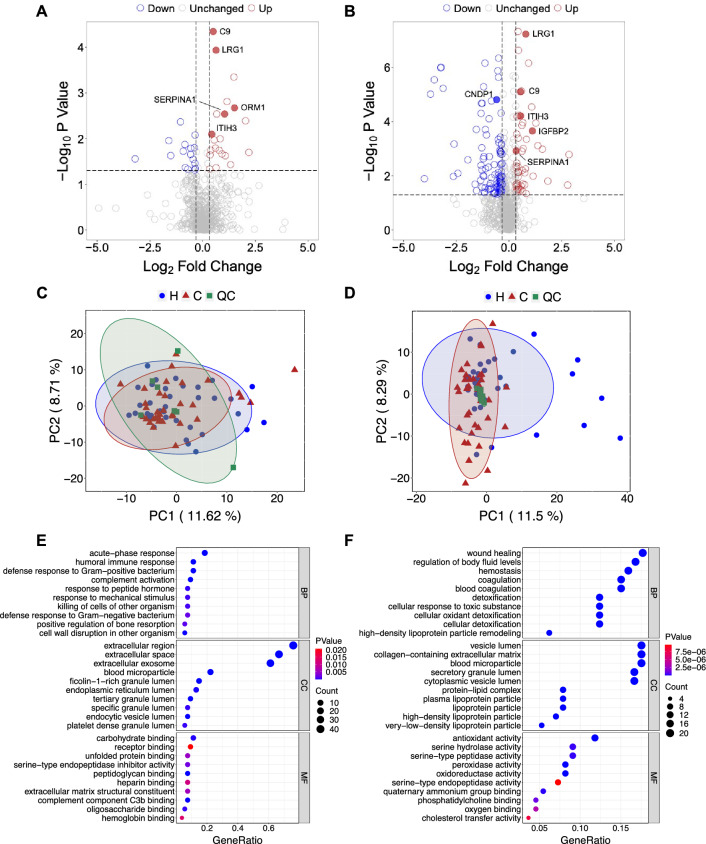
Quantitative proteomic analysis of plasma samples from CRC patients and healthy controls. **A** Volcano plot of DDA data obtained from discovery cohort 1. Differentially expressed proteins are shown in blue (down) or red (up) circles. X-axis shows log2-fold change of plasma proteins between CRC patients and healthy subjects, and y-axis shows log10 of statistical significance values. **B** Volcano plot of DIA data obtained from discovery cohort 2. The label for the differentially expressed proteins and the two axes are the same as in A. **C** Principal component analysis of the protein expression data in cohort 1. **D** Principal component analysis of the protein expression data from cohort 2. **E** Gene ontology analysis of up-regulated proteins in the DIA data. **F** Gene ontology analysis of down-regulated proteins in the DIA data

The QC samples showed good correlation (and thus reproducibility) in DDA data among themselves in a pair-wise comparison matrix, as shown by the Pearson correlation in Additional file 1: Fig. S1A. The correlation was even better in DIA data as shown by the correlation matrix (Additional file 1: Fig. S1B), indicating that DIA provide more overall accuracy and consistency than DDA as have been shown previously [25]. Both DDA and DIA covered a dynamic range of 6 order of magnitude, with DIA covered a wider dynamic range (Additional file 1: Fig. S1C–D). Furthermore, DIA quantified more proteins than DDA in all samples, as shown in Additional file 1: Fig. S1E–F. We quantified 607 proteins in DDA experiments and 714 proteins in DIA experiments (Additional file 7: Table S2 and Additional file 8: Table S3). Using a statistical significance cutoff of 0.05 and fold change cutoff of 1.25, we identified 21 up-regulated and 16 down-regulated proteins in DDA experiments, and 50 up-regulated and 106 down-regulated proteins in DIA experiments as shown in the volcano plots (Fig. 2A, B). Principal component analysis using the entire proteomic data failed to separate the disease, healthy and the QC samples, neither in the DDA data (Fig. 2C) nor in the DIA data (Fig. 2D). In up-regulated proteins, gene ontology of the most enriched biological function was primarily acute-phase response and humoral immune response, with the corresponding KEGG pathways involved being complement and coagulation cascades (Fig. 2E and Additional file 2: Fig. S2). Whereas in down-regulated proteins, the most enriched biological function was wound healing which involves remodeling of the extracellular matrix, and the corresponding KEGG pathways involved were carbon metabolism and biosynthesis of amino acids (Fig. 2F and Additional file 2: Fig. S2). Several important intracellular signaling pathways including Wnt signaling, PI3K-Akt signaling, and p53 signaling are frequently dysregulated in CRC [26–28]. A recent study demonstrated that CRC cell survival was related to an impaired hypoxia-inducible factor 1-alpha (HlF-1a) signaling in low oxygen condition [29]. Among these important driving events of tumorigenesis, some were captured in our findings [27, 28].

### Discovery of a plasma protein biomarker panel to identify CRC at early stages

**Fig. 3:**
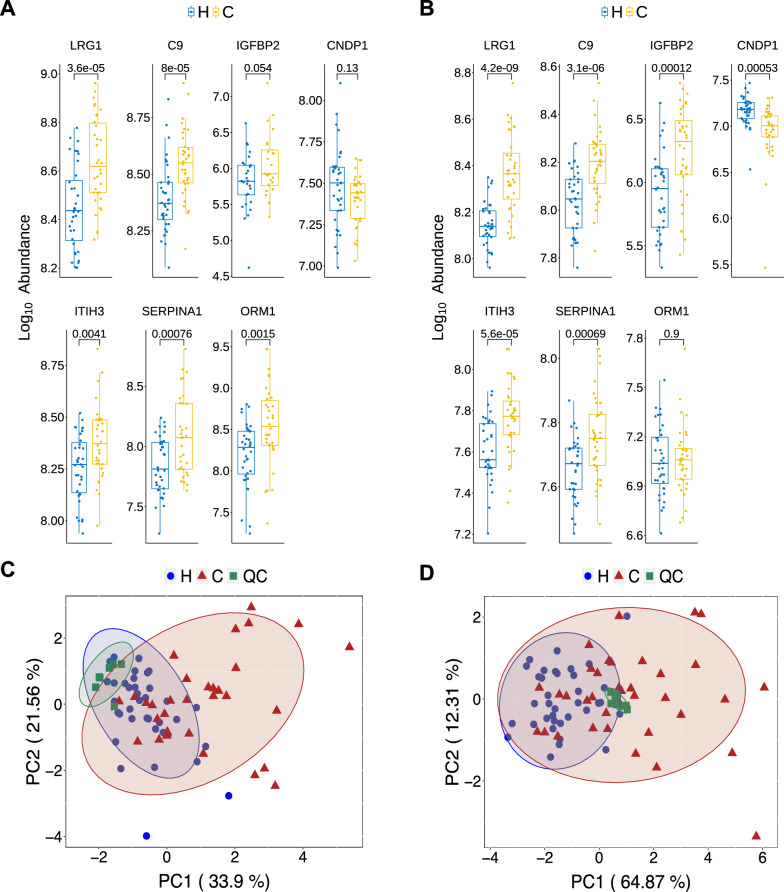
Seven feature proteins selected based on DDA and DIA proteome data. **A** and **B** Boxplot of protein intensity of feature proteins selected for logistic regression. Differential expression of seven proteins between CRC patients (**C**) and healthy subjects **H** from the DDA data (**A**) and the DIA data (**B**) are shown. **C** Principal component analysis using the expression levels of the seven proteins in cohort 1. **D** Principal component analysis using the expression levels of the six proteins in cohort 2

To identify biomarkers in the plasma that can accurately distinguish early-stage CRC from healthy subjects, we selected the top 40 proteins based on MDG values (Additional file 3: Fig. S3). From these proteins we identified consistent up-regulation of LRG1, C9, IGFBP2, ITIH3, and SERPINA1 and consistent down-regulation of CNDP1 in CRC plasma as potential biomarker candidates (Fig. 3A, B). ORM1 was found in DIA data but did not show differential expression; however, it was up-regulated in the DDA data (Fig. 3A). This discrepancy maybe due to variation in mass spectrometric data acquisition. Because ORM1 has been shown to be a biomarker for CRC [30, 31], we added it to the potential list of our biomarker panel. The peptide sequences of the panel of seven proteins were listed in Additional file 4: Fig. S4. Using the expression data of these seven proteins, principal component analysis (PCA) showed much improved separation between CRC and healthy subjects, with the first component explained 33.9% (DDA data) and 64.8% (DIA data) of the variability (Fig. 3C, D).

### The plasma protein biomarker panel is capable of detecting colorectal cancer with high accuracy

Because DDA and DIA are relative quantification methods and MRM is able to measure the absolution concentration of an analyte with higher throughput, we developed an MRM assay using signature peptides to measure the concentration of the seven proteins in the plasma, in a subset of 60 healthy subjects and 60 CRC patients from the discovery cohort (Fig. 1B). The MRM assay combined internal standards using heavy arginine/lysine-labeled peptides with external calibration using unlabeled peptides. The product ion peak of each peptide appeared superior in our MRM assay (Additional file 5: Fig. S5A–D) with the majority of the covariance (CV) of the measured peptide concentration below 10% (Fig. 5E). Using concentrations of the seven proteins to build a logistic regression model, we calculated a classification score for each patient. The score was calculated using the following equation:

log(P/(1-P) = -7.8709 + 4.5956 × IGFBP2 + 0.2732 × ITIH3 + 0.0909 × LRG1 + 0.1015 × C9 -3.2205 × CNDP1 + 0.0239 × ORM1 + 0.0811 × SERPINA1, where P is a value between 0 and 1 that represents the probability of the event (colorectal cancer). The optimal cut-off value for the classifier was 0.318.

**Fig. 4:**
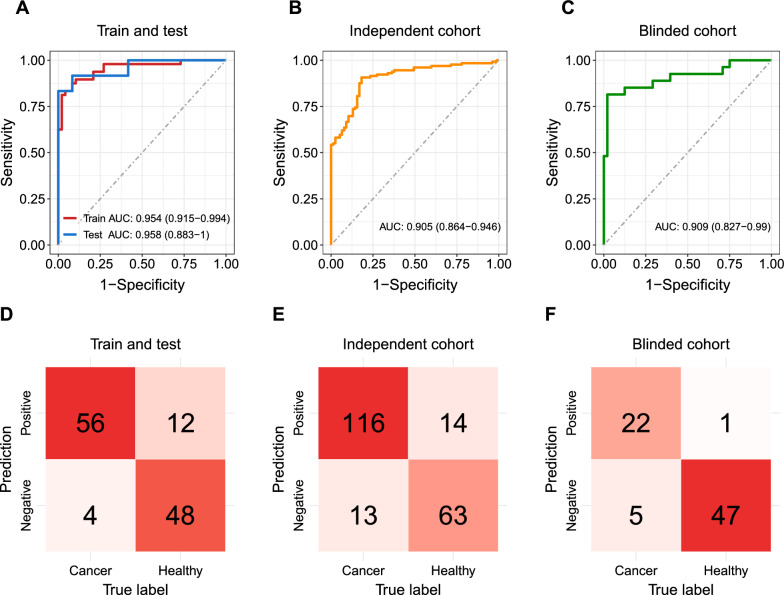
MRM quantification and logistic regression classification of CRC and healthy subjects. **A** ROC curves of a seven-protein logistic regression classifier (LRG1, C9, IGFBP2, CDNP1, ITIH3, SERPINA1, and ORM1) for distinguishing CRC and healthy subjects in the training and testing datasets. **B** ROC curve showing the performance of the seven-protein classifier in distinguishing CRC and healthy subjects in an independent validation cohort. **C** ROC curve showing the performance of the seven-protein classifier in distinguishing CRC and healthy subjects in a blinded validation cohort. **D**–**F** Confusion matrix showing the classification accuracy in the training (**D**), independent validation **E**, and blinded validation **F** cohorts

This logistic regression classifier achieved an average area under the curve (AUC) of the receiver operator characteristic (ROC) curve of 0.954 (95% CI 0.915–0.994) in the training dataset and 0.958 (95% CI 0.883–1.0) in the testing dataset (Fig. 4A). Furthermore, we applied the MRM assay to quantify the concentration of the seven proteins in plasma samples collected from an independent validation cohort from different hospitals (Additional file 9: Table S4). Applying the locked logistic regression parameters to calculate the likelihood of CRC and the fixed cutoff probability value of 0.318, we achieved an average AUC of 0.905 (Fig. 4B, 95% CI 0.864–0.946). We enrolled another independent validation cohort in a blinded fashion, in which the diagnosis of the patients was disclosed only after the classification was determined (Additional file 9: Table S4). We achieved an average AUC of 0.909 using the same assay and the locked logistic regression parameters and probability cutoff value (Fig. 4C, 95% CI 0.827–0.99).

Based on the confusion matrix, the assay had a sensitivity, specificity, PPV, and NPV of 93.3%, 80.0%, 82.4%, and 92.3% respectively in the training cohort (Fig. 4D), and 89.9%, 81.8%, 89.2%, and 82.9% respectively in the validation cohort (Fig. 4E). In the blinded validation cohort, the sensitivity, specificity, PPV, and NPV were 81.5%, 97.9%, 95.6%, and 90.4% respectively (Fig. 4F); the classification accuracy was 92.0% (Table 2B). Overall, the sensitivity of detecting CRC achieved 91.0% with a specificity of 81.0% (Table 2A).

**Table 2:** Sensitivity and specificity of the protein classifier panel for detecting colorectal cancer and advanced precancerous lesions

	Colonoscopy	7-Protein biomarker	
A		Positive result	Sensitivity
Cancer (CRC)	189	172	91.0%
Advanced precancerous lesions (APC)	47	23	49.0%
			Specificity
Negative (H)	137	26	81.0%
B		Positive result	Sensitivity
Cancer (CRC)	27	22	81.5%
High-grade dysplasia	9	4	44.4%
			Specificity
Negative (H)	48	1	97.9%

### Expression patterns of the seven protein biomarkers at different CRC stages

**Fig. 5:**
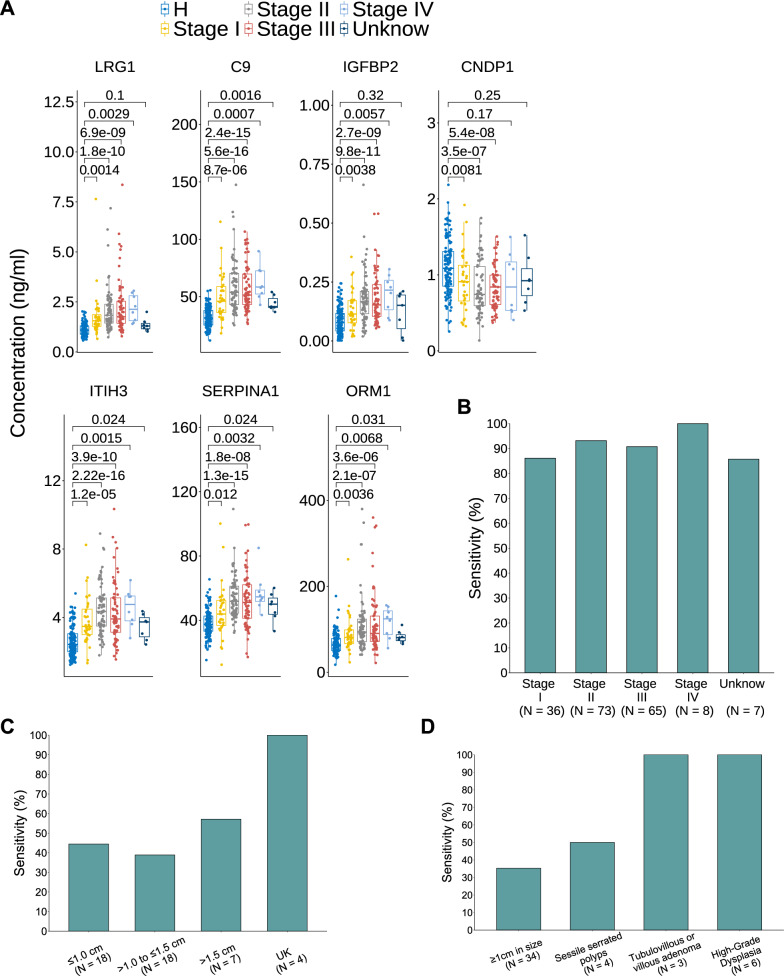
Expression patterns of the protein biomarkers in different CRC stages. **A** Box plot of plasma concentration of the seven proteins in four different CRC stages. **B** Sensitivity of the seven-protein biomarker in distinguishing CRC at four different stages from healthy subjects. **C** Sensitivity of the seven-protein biomarker in distinguishing APC with different sizes from healthy subjects. **D** Sensitivity of the seven-protein biomarker in identifying precancerous lesions with different grades

We further analyzed the capability of the seven-protein panel to distinguish CRC at different stages. In general, each individual protein showed significantly different expression levels in every pathological stage compared to healthy subjects; however, there were no obvious statistical difference between stages II ~ IV (Fig. 5A). Nevertheless, there were significant differences between later stages and stage I in proteins IGFBP2, C9, SERPINA1, and ORM1 (Additional file 10: Table S5). The sensitivity of our protein panel reached over 90% in detecting CRC at all stages; however, there was a clear pattern of increased sensitivity in later stages (II ~ IV) than stage I (Fig. 5B). The sensitivity of detecting APC was around 40% for lesions smaller than 1 cm or between 1 and 1.5 cm, but increased to nearly 60% for lesions greater than 1.5 cm (Fig. 5C). This result showed an increased sensitivity in detecting APC than a published DNA test [14]. Finally, the sensitivity of detecting colorectal polyps of distinct anatomic morphology was 50% for sessile serrated polyps, 100% for tubulovillous adenoma and high-grade dysplasia (Fig. 5D), all of which are considered as having high probability of transforming into cancer. Thus, our panel of protein biomarkers can not only detect CRC at early stages, but also detect highly malignant adenomas.

## Discussion

The biological functions of many of the biomarker proteins identified in this study have been documented. Overall, they play distinct roles in cancer development and progression. For example, LRG1 is a cell adhesion molecule whose up-regulation is involved in tumor metastasis [32], and has recently been shown to be valuable in diagnosis of colorectal cancer and pancreatic cancer [16, 33]. As a complement component that is linked to inflammation and immune response, C9 is unlikely to provide specific information predicting tumor formation. Nevertheless, a recent study found aberrant expression of C9 in the plasma of CRC patients [34]. IGFBP2 is a node of insulin signaling and has been shown to have prognostic value in multiple cancers in a meta-analysis [35], including metastatic CRC [36]. Similarly, ORM1, SERPINA1, and ITIH3 has all been shown to be valuable predicting clinical outcomes of CRC patients [31, 37, 38]. Down-regulation of CNDP1 was associated with cancer cachexia [39], and it was recently reported that CNDP1 level was significantly reduced in hepatocellular carcinoma tissues [40]. Although these studies focused on different cancer types, they all point to CNDP1 down-regulation in cancers, which are consistent with our study. By accurately measuring the plasma concentration of these proteins, we could predict CRC at an early stage using logistic regression.

There have been numerous attempts to develop protein-based liquid biopsy assays for detection of CRC. A noticeable study used proximity extension assay (Olink) to measure plasma concentration of 92 proteins from 89 subjects, and identified an eight-protein biomarker with an adjusted AUC of 0.77 and a sensitivity of 0.44 at 90% specificity in the validation set [41]. Because of the high sensitivity of Olink technology [42], it is able to quantify plasma concentration of very-low abundant proteins such as growth factors, cytokines, and tumor antigens, none of which overlapped with our panel components. However, the resulting performance of predicting CRC remained unsatisfactory, presumably due to the overfitting issue of measuring large number of proteins. Another study used mass spectrometry to identify a five-protein biomarker signature to effectively distinguish CRC from control in a training cohort of 200 cases and a validation cohort of 269 cases, with an AUC of 0.84 and an overall accuracy of 72% [43]. Of note is that the five-protein panel includes LRG1, one of the feature protein in our seven-protein panel. Another protein in this five-protein panel is SERPINA3, which is a close relative to SERPINA1 in our seven-protein panel. This indicates that mass spectrometry tends to detect proteins at similar expression levels. Compard to these studies, our assay of seven-protein panel resulted an AUC of 0.905 and 0.909 in two independent validation cohorts, providing a much-improved performance. Even compared to a study that combines DNA test and fecal immune test which provided a sensitivity of detecting CRC at 92.3% and specificity of 86.6% [14], our assay provides a slightly lower but comparable sensitivity (91.0%) and specificity (81.0%) (Table 2A). In addition to detecting cancers, there is a growing need to identify colorectal adenomas using protein biomarkers in the blood. A recent study found that serum biomarkers F5, ITIH4, LRG1, and VTN were elevated in colorectal adenoma patients as well as in a mouse model of colorectal adenomas [16]. From colonoscopy-confirmed patients with advanced precancerous lesions, the average sensitivity of our assay achieved 49% (Table 2B), better than aforementioned study. Our assay also showed a clear improvement compared to the DNA test which provided a sensitivity of 42.4% to detect APC [14].

From our quantitative proteomic results, it appears that DDA has more upregulated proteins and DIA has more of downregulated proteins. Because DDA is data dependent, it favors detection and quantification of relatively abundant peptides. DIA is utilizing homogeneous scanning over the entire mass range with a wider mass window on each scan, and detects and quantifies peptides regardless of their abundance. Therefore, DIA is capable of detecting low abundance peptides and quantifying more proteins. It is also possible that the differentially expressed proteins in DDA and DIA experiments might distribute with different patterns. Regardless, six out of the seven significantly changed marker proteins in both DDA and DIA experiments showed consistent direction of change (Fig. 3A, B). We believe that DDA and DIA are complementary, and there no black-and-white answer as to which one is more trustworthy over the other. Largely due to the stochastic nature of mass spectrometry data acquisition, quantifying large number of peptides may results in certain degree of error rate.

For the profiling experiments in discovery phase, we took the conventional approach to remove the 14 most abundant plasma proteins using the antibody-based depletion kit. Depletion of abundant proteins can reduce the interference from these proteins during LC–MS/MS data acquisition and improve the depth of the coverage of the plasma proteome [44]. However, because it introduces more sample processing steps, it considerably affects the reproducibility of the experiment. This may be further exaggerated when dealing with large number of clinical samples. Therefore, during target validation phase of the MRM assay development, we innovatively simplified the sample preparation process by omitting the depletion step. Of the proteins we measured, the plasma concentrations ranged between 1 ng/ml for CNDP1 and 120 ng/ml for ORM1, and we obtained decent product ion signals in all seven peptides (Additional file 5: Fig. S5A–D). The results also point to acceptable precision in clinical settings even though the experiments were performed manually, as demonstrated by lower than 10% CV in QC samples for the majority of the proteins measured (Additional file 4: Fig. S4E). Only the concentration of IGFBP2 showed a CV of over 10%, presumably due to its extremely-low abundance. On the other hand, ORM1 is one of 14 high-abundance proteins targeted for depletion; removing it runs the risk of removing a potentially meaningful biomarker candidate. With our simplified sample processing method, we envision that by incorporating automation in the future, we will further improve the precision and accuracy of our measurement.

The strength of this study lies in several technical and strategical advantages. First, many previous CRC protein biomarker studies started from comprehensive literature search for candidate proteins, and then developed quantitative assays to build models and further validated the model. This approach may be difficult to discover new biomarkers. In our study, we started from an unbiased quantitative analysis of plasma proteome using the state-of-art mass spectrometry technology to discover candidate proteins; this study design facilitated MRM assay development in the validation phase. Second, after establishing a machine learning model based on the training cohort of 120 subjects, we enrolled two independent patient cohorts of 253 and 84 subjects from three different hospitals to validate the model. In particular, the second validation cohort were performed in a blinded fashion, moving one step closer to a prospective study. Noticeably, in protein-based biomarker studies, the cohort size in our study is reasonably large. Third, when applying the MRM assay and the “locked” logistic regression model parameters on both independent validation cohorts, our assay maintained an accuracy of over 90%. Taking into consideration the CRC prevalence of around 0.25% in Chinese population [45] with the sensitivity of 81.5% and specificity of 97.9%, the calculated NPV of our assay can reach 99.95%. The sensitivity and specificity of our assay compare favorably to existing clinical diagnosis and screening methods including stool FOBT/FIT [5], blood and stool DNA methylation test [6, 7], and performs similarly to an FDA approved, stool-based DNA test (Cologuard) [14], well suited for early screen. Fourth, because sampling blood is more convenient than procuring stool samples and its non-invasive nature surpasses colonoscopy, patient compliance is expected to be much better than conventional diagnostic methods. If applied as an early screening method, it can prevent a significant number of medium-to-high risk subjects from invasive procedures. Finally, our plasma sample processing approach omitted the use of expensive depletion kit, which is not only technically advantageous but also has the benefit of significantly cutting the cost of the assay. This advantage could be further augmented if the assay is applied in settings involving population screen.

One limitation of this study is the relatively sub-optimal sample size. Another limitation is that the subjects were recruited from hospital patients; whereas in a real-world screening test, the subjects would come from the population undergone preventive physical examination and thus the number of cancer patients would have been much less. Further refinement and validation of this panel of biomarker proteins in a population-wide scenario for a clinical trial is warranted.

## Conclusion

We have developed a non-invasive, targeted mass spectrometry assay to measure plasma concentrations of seven proteins that is capable of distinguishing colorectal cancer from healthy subjects. This seven-protein classifier is of translational value and warrants further development into a clinically useful assay.

### Supplementary Information

**Additional file 1: Figure S1. **Quality of the DDA and DIA data. (A) Correlation analysis of the quality control (QC) samples from DDA data. (B) Correlation analysis of the quality control (QC) samples from DIA data. (C) Distribution of protein abundance of all quantified proteins from DDA data. (D) Distribution of protein abundance of all quantified proteins from DIA data. (E) Number of quantified proteins in all samples from DDA data. (F) Number of quantified proteins in all samples from DIA data.

**Additional file 2: Figure S2. **Heatmap showing KEGG pathways of up- and down-regulated proteins in the plasma from CRC patients.

**Additional file 3: Figure. S3. **Selection of protein panels to classify CRC from healthy subjects. (A) Mean decrease of Gini index in DDA data. (B) Mean decrease of Gini index in DIA data.

**Additional file 4: Figure S4. **Peptide sequence and ion mass information of selected protein markers.

**Additional file 5: Figure S5. **Quality of the MRM data. (A–D). Extracted chromatograms of the 7-peptide biomarkers. (E) Variable coefficient values in QC samples for the 7-peptide biomarkers.

**Additional file 6: Table S1. **Demographic data of all the patients enrolled in MRM experiments.

**Additional file 7: Table S2. **Protein expression data from DDA experiments.

**Additional file 8: Table S3. **Protein expression data from DIA experiments.

**Additional file 9: Table S4. **Concentration of each protein of the seven-protein panel measured by MRM in all subjects.

** Additional file 10: Table S5. **Concentration and statistical analysis of each protein of the seven-protein panel measured by MRM in CRC cases shown in Figure 4, D and E.

## Data Availability

The datasets generated (mass spectrometry raw data) during the current study has been deposited to the ProteomeXchange repository with the accession numbers PXD042639 and PXD042652 (https://proteomecentral.proteomexchange.org/cgi/GetDataset). The analyzed data is included in the supplementary tables. The clinical samples and analytical methods will not be available to the public.
