# Supplementary figures and images for: A proteomic classifier panel for early screening of colorectal cancer: a case control study
Source: J Transl Med. 2024 Feb 21;22:188. doi: 10.1186/s12967-024-04983-5 (PMC10880210; doi:10.1186/s12967-024-04983-5)

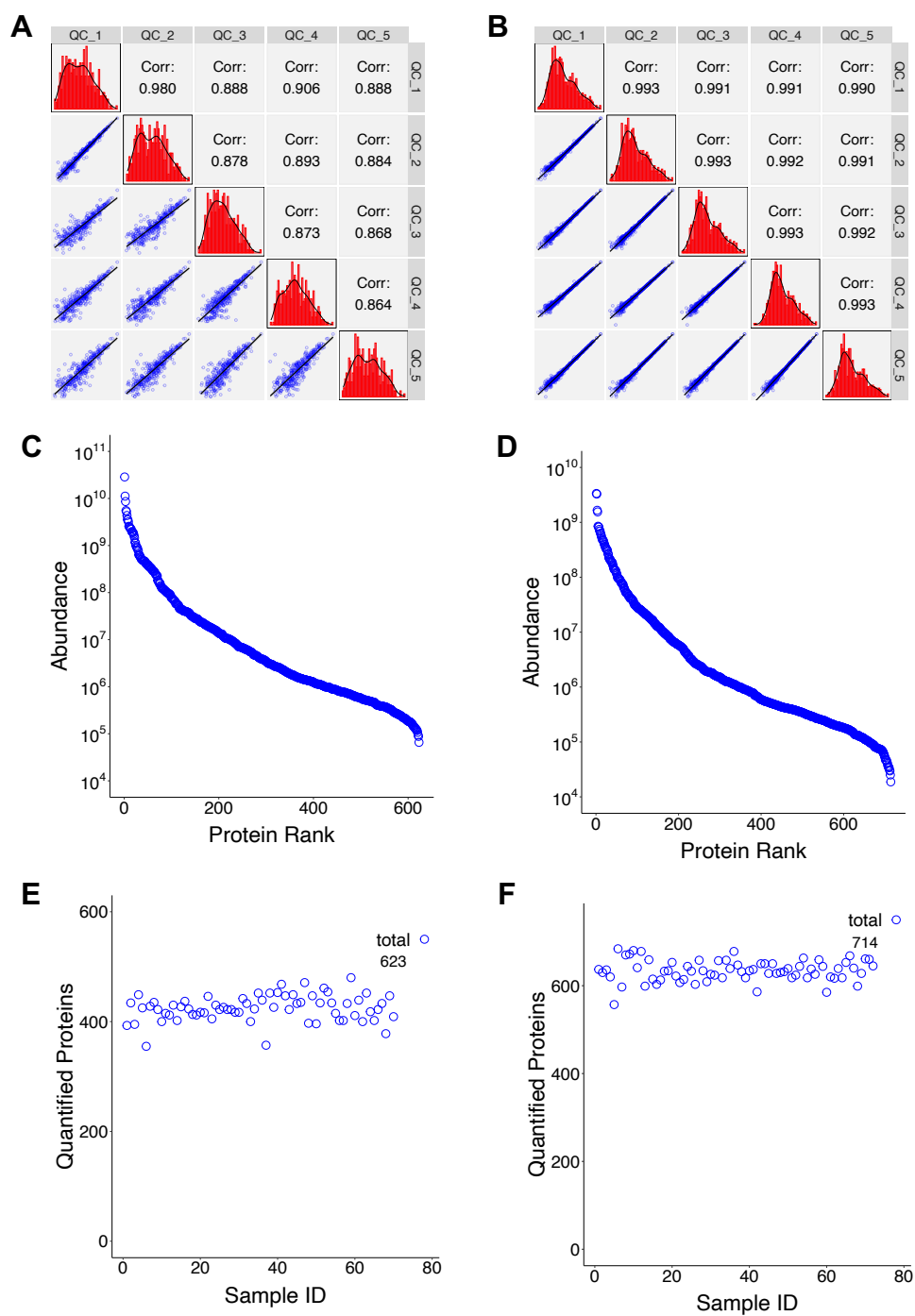

Supplement: Supplementary file 1 — Additional file 1: Figure S1. Quality of the DDA and DIA data. (A) Correlation analysis of the quality control (QC) samples from DDA data. (B) Correlation analysis of the quality control (QC) samples from DIA data. (C) Distribution of protein abundance of all quantified proteins from DDA data. (D) Distribution of protein abundance of all quantified proteins from DIA data. (E) Number of quantified proteins in all samples from DDA data. (F) Number of quantified proteins in all samples from DIA data. [file 12967_2024_4983_MOESM1_ESM.pdf]

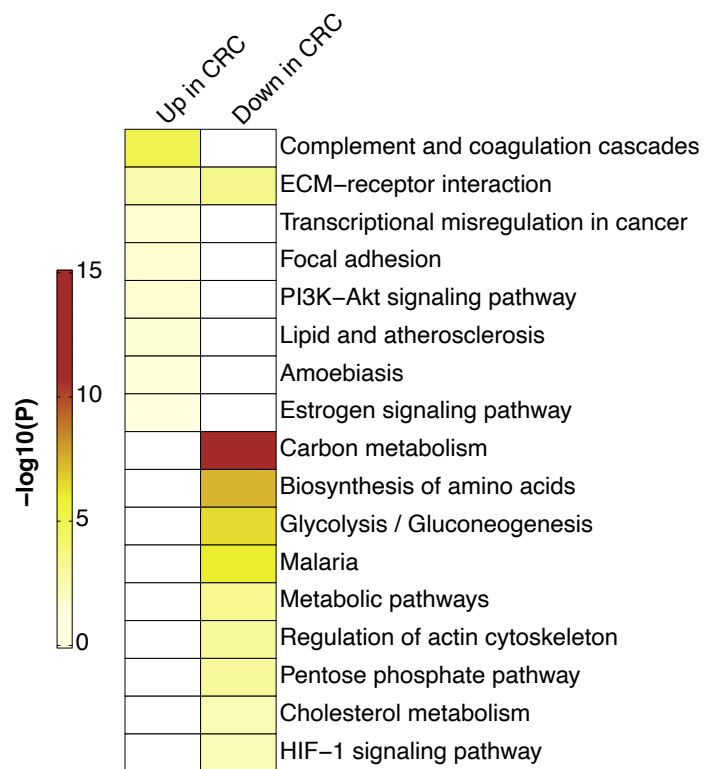

Supplement: Supplementary file 2 — Additional file 2: Figure S2. Heatmap showing KEGG pathways of up- and down-regulated proteins in the plasma from CRC patients. [file 12967_2024_4983_MOESM2_ESM.pdf]

A

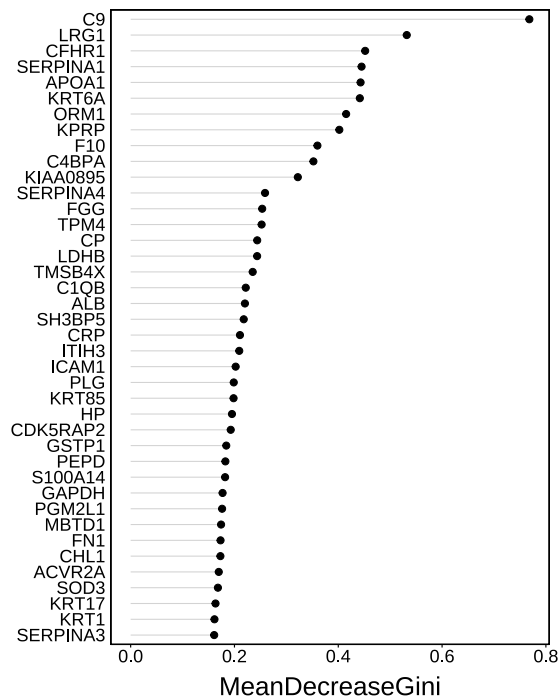

B

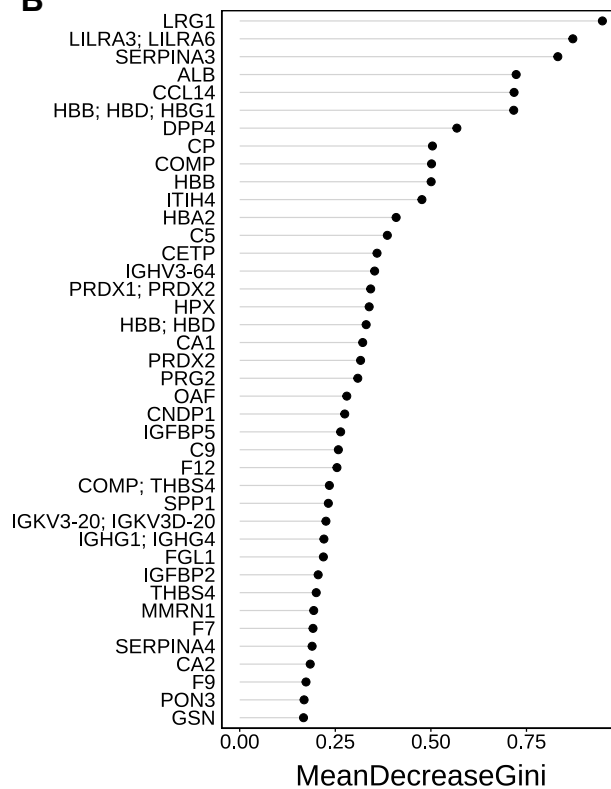

Supplement: Supplementary file 3 — Additional file 3: Figure. S3. Selection of protein panels to classify CRC from healthy subjects. (A) Mean decrease of Gini index in DDA data. (B) Mean decrease of Gini index in DIA data. [file 12967_2024_4983_MOESM3_ESM.pdf]

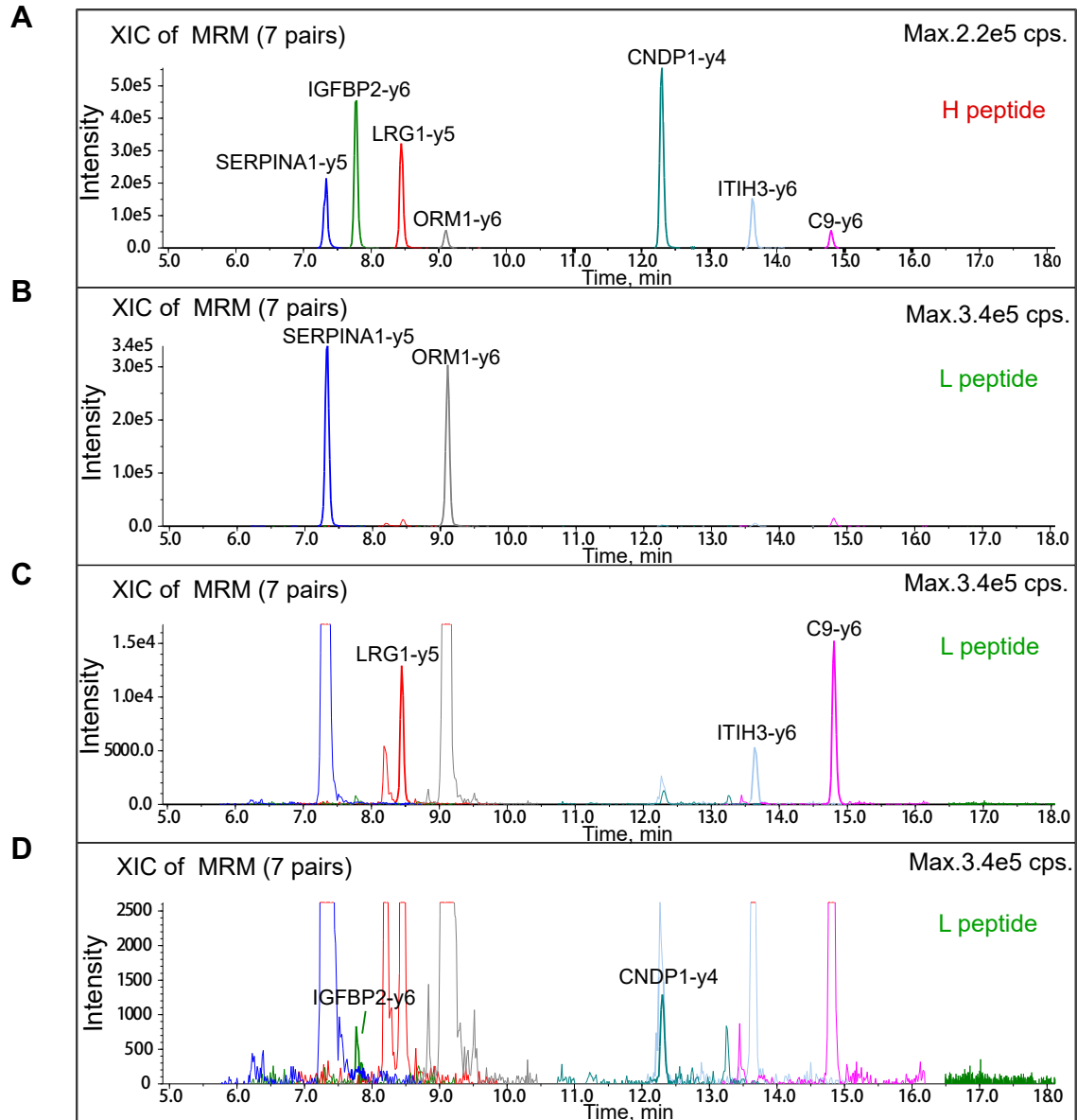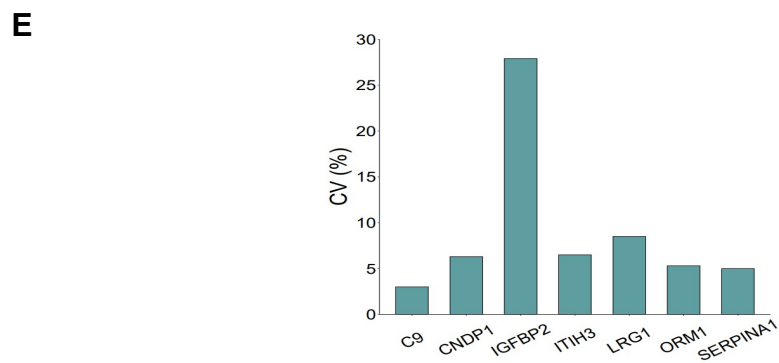

Supplement: Supplementary file 5 — Additional file 5: Figure S5. Quality of the MRM data. (A–D). Extracted chromatograms of the 7-peptide biomarkers. (E) Variable coefficient values in QC samples for the 7-peptide biomarkers. [file 12967_2024_4983_MOESM5_ESM.pdf]
