# Supplementary material for: A proteomic classifier panel for early screening of colorectal cancer: a case control study
Source: J Transl Med. 2024 Feb 21;22:188. doi: 10.1186/s12967-024-04983-5 (PMC10880210; doi:10.1186/s12967-024-04983-5)
Supplement: Supplementary file 4 — Additional file 4: Figure S4. Peptide sequence and ion mass information of selected protein markers. [file 12967_2024_4983_MOESM4_ESM.pdf]

Protein name	Peptide sequence	Charge	Precursor Mass	Product ion Mass
IGFBP2	LIQGAPTIR	2	484.8	614.4
ITIH3	DYIFGNYIER	2	645.3	751.4
LRG1	GQTLLAVAK	2	450.8	501.3
C9	AIEDYINEFSVR	2	728.4	751.4
SERPINA1	AVLTIDEK	2	444.8	605.3
CNDP1	EWVAIESDSVQPVPR	2	856.44	468.29
ORM1	SDVVYTDWK	2	556.8	811.4
